# Associations between microaggressions, depression, anxiety, and alcohol use among Black young adults: findings from a pilot study

**DOI:** 10.3389/fpubh.2025.1694000

**Published:** 2026-01-15

**Authors:** Patricia A. Cavazos-Rehg, Xiao Li, Layna Paraboschi, Lucy Meigs, Erin Kasson, Hannah S. Szlyk, JaNiene E. Peoples, Devin E. Banks, Alex T. Ramsey, Nicholas C. Jacobson, Carolyn E. Sartor

**Affiliations:** 1 Department of Psychiatry, Washington University School of Medicine, St. Louis, MO, United States; 2 College of Social Work, Florida State University, Tallahassee, FL, United States; 3 Center for Technology and Behavioral Health, Geisel School of Medicine, Dartmouth College, Hanover, NH, United States; 4 Institute for Health, Health Care Policy, and Aging Research, Robert Wood Johnson Medical School, Rutgers University, New Brunswick, NJ, United States

**Keywords:** alcohol, Black/African American, young adults, discrimination, depression

## Abstract

**Objective:**

This pilot study, which was designed to inform mobile health intervention development, assessed the frequency of various microaggressions and explored associations among microaggressions, alcohol use, depression, and anxiety in Black young adults.

**Methods:**

Ninety-two Black adults (mean age = 22.2 [SD = 1.9]) who regularly consume alcohol were recruited through social media to complete a survey on microaggressions, depression, anxiety, and alcohol use. Microaggression frequencies were calculated and a Cochran–Mantel–Haenszel test was used to model interrelationships among microaggressions, depression, and anxiety with respect to binge drinking.

**Results:**

Past-30-day microaggression experiences were reported by 82.6% of participants. Stratified analyses signaled that the association between moderate/severe depression and binge drinking exclusively among individuals who had past-30-day microaggression experiences, approached statistical significance (OR = 2.58, 95% CI:0.94, 7.01; Cohen’s *d* = 0.52).

**Conclusion:**

Findings highlight the pervasiveness of microaggressions and suggest that they may play a key contextual role in shaping binge drinking behaviors among Black young adults experiencing depression.

## Introduction

Black young adults frequently encounter day-to-day discrimination experiences, referred to as microaggressions, based on race or other aspects of identity [e.g., gender (1)]. They are also at high risk for experiencing alcohol related harms (2, 3). Further, rates of depression and anxiety in this population are increasing (4, 5). The association of racial discrimination with problem drinking is well documented (6, 7) and there is evidence for increased symptoms of depression and anxiety among Black young adults who experience high levels of racial discrimination (8, 9), including microaggressions (10).

Ecological momentary assessment (EMA) provides a means of exploring these phenomena in real time. EMA studies assessing racial microaggressions in Black adults have revealed the environments in which they most commonly occur and linked microaggressions to depression symptoms (11). Moreover, mobile health (mHealth; i.e., smartphone app-based) interventions have demonstrated efficacy and acceptability among Black young adults, including a preference for culturally responsive content addressing the unique experience of this population; however, more research is needed to optimize these interventions (12). The current pilot study was designed to lay the groundwork for an EMA data collection and subsequent development of an mHealth intervention addressing alcohol use and mental health impacts of microaggressions in Black young adults. The aims were to explore (1) how often and in what ways Black young adult drinkers experience microaggressions and (2) the association of microaggressions with alcohol use, depression, and anxiety symptoms in this population.

## Methods

### Procedures

Participants were recruited using social media advertisement campaigns targeted toward Black/African American young adults, managed by BuildClinical (13). Respondents who clicked on these ads were routed to additional study information and, if interested, could consent and complete the online eligibility screener. Study eligibility criteria were: (1) 18–25 years old, (2) self-identified as Black or African American, (3) U.S. resident, (4) English speaking, and (5) consuming alcohol on average two or more days per week over the last 3 months. Participants completed a one-time survey; a subset subsequently provided feedback on an EMA app. The current study reports results of the survey, which informed design parameters for the EMA component.

Data monitoring steps, consistent with best practices for fraud prevention in online surveys (14) were implemented. Respondents deemed ineligible or potentially fraudulent received a discharge email containing nationwide alcohol use and mental health resources. Eligible respondents (1) received a follow-up phone call to verify that they were not a bot or duplicate screen and to confirm screen answers; (2) were provided a personalized link to an online consent form; and (3) proceeded to the survey. All online consent procedures and surveys were completed in REDCap. The Washington University Institutional Review Board approved the study (202212052) and categorized it as minimal risk.

Of 1,925 screened participants, 133 completed the survey. Exclusions included invalid responses to the alcohol use assessment (e.g., binge drinking days exceeding total drinking days; *n* = 27), and missing data on key assessments (e.g., depression measure; *n* = 14), yielding a final sample size of 92.

### Measures

#### Demographic characteristics

Age, sex assigned at birth, gender identity, student status, community type (e.g., urban), and three indicators of socioeconomic status (access to basic needs, educational attainment of participant, and educational attainment of primary caregiver) were queried.

#### Past 30-day binge drinking status (no/yes)

Past-30-day binge drinking was assessed by asking, “Considering all types of alcoholic beverages, how many days during the past 30 days did you have 5 or more drinks (men)/4 or more drinks (women) on a single occasion?” Reports of one or more days were coded “yes.”

#### Microaggressions

The Everyday Discrimination Scale (EDS) is a 9-item measure assessing day-to-day experiences of discrimination (i.e., microaggressions). Items include “People act as if they are afraid of you” and “You are treated with less respect than other people are.”) (15). EDS items have been used in previous EMA studies to capture discrimination experiences (16, 17). Respondents rate each item on a scale from 0 (never) to 5 (almost every day). Item values are summed, with a possible range of 0–45 (*α* = 0.89 in the current sample). Given our interest in capturing microaggressions experienced by Black young adults with respect to any aspect of identity, questions were not specific to experiences attributable to race. Dichotomous indicators of any microaggression experiences across the lifetime (i.e., 0 (never) vs. 1–5 (less than once a year – almost every day) and in the past 30 days (i.e., 0–2 (never to a few times a year) vs. 3–5 (a few times a month to almost every day)) were also generated. These indicators were used to distinguish participants with frequent recent microaggressions from those with less frequent or no recent experiences, providing simple risk groups that are relevant for intervention planning.

#### Depression

The Patient Health Questionnaire (PHQ-9) was used to assess symptoms of depression over the past 2 weeks [removing the item assessing suicidality (18)]. Participants reported the frequency of each symptom on a scale from 0 (not at all) to 3 (nearly every day). The score was calculated by summing (*α* = 0.88 in the current sample). Scores ≤ 9 were categorized as none or mild; scores ≥10 were categorized as moderate or severe depression (19). This categorization facilitated interpretation in terms of clinically meaningful symptom burden and reduced model complexity in this pilot sample.

#### Anxiety

The seven-item Generalized Anxiety Disorder Scale (GAD-7) was used to assess symptoms of anxiety over the past 2 weeks. Item responses ranged from 0 (not at all) to 3 (nearly every day), and the score was generated by summing (*α* = 0.91 in the current sample). A cut-off point of 9 was used to dichotomize the results into none or mild (≤9) versus moderate or severe (≥10) anxiety (20) to reflect clinically relevant symptom levels and to limit the number of parameters estimated in this small pilot sample.

### Statistical analysis

Descriptive statistics (i.e., means, counts, and proportions) were conducted to characterize the sample, including demographics, prevalence of microaggressions, prevalence of binge drinking, and levels of depression and anxiety. We used the Cochran–Mantel–Haenszel (CMH) test to examine whether associations between depression (none/mild vs. moderate/severe) or anxiety (none/mild vs. moderate/severe) and binge drinking differed by recent microaggression experiences (yes/no). The CMH test is a means of assessing conditional associations in small samples without adding model complexity. It provides stratum-specific and common odds ratios, allowing for a direct comparison of associations across microaggression strata (21–23). In this pilot sample, CMH offered a more stable and transparent approach than logistic regression models that would require additional terms (e.g., microaggressions × depression/anxiety interactions). Statistical significance of a Breslow-Day test for homogeneity indicates the presence of a common OR. Both conditional and common ORs are reported, along with their corresponding 95% confidence intervals (CIs). Statistical analyses, performed in SAS Version 9.4., were two-sided.

## Results

Sample characteristics are reported in Table 1. The majority of participants reported binge drinking in the past 30 days (68.5%). Moderate to severe depression was endorsed by 48.9%, moderate to severe anxiety by 50.0% of the sample.

**Table 1:** Sample characteristics (*N* = 92).

	Overall *N* (%)
Age, Mean (SD)	22.2 (1.9)
Sex at birth	
Female	47 (51.7)
Male	45 (48.3)
Gender identity	
Cisgender woman	40 (43.5)
Cisgender man	43 (46.7)
Transgender man	3 (3.3)
Transgender woman	0
Genderqueer/Gender nonconforming/Prefer to self-identify	5 (5.4)
No response	1 (1.1)
Student status	
Full-time student	44 (47.8)
Part-time student	6 (6.5)
Non-student	42 (45.7)
Participant’s educational attainment	
Below bachelor’s degree	47 (51.7)
Bachelor’s degree or higher	45 (48.3)
Caregiver’s educational attainment	
Below bachelor’s degree	36 (39.1)
Bachelor’s degree or higher	55 (59.8)
Do not know	1 (1.1)
Meeting basic needs	
Very hard/hard/somewhat hard	35 (38.0)
Not very hard	57 (62.0)
Type of community of residence	
Urban	56 (60.9)
Suburban	34 (36.9)
Rural	1 (1.1)
Do not know	1 (1.1)
Binge drinking alcohol in past 30 days	63 (68.5)
Depression	
None/mild	47 (51.1)
Moderate/severe	45 (48.9)
Anxiety	
None/mild	46 (50.0)
Moderate/severe	46 (50.0)
Experienced microaggression in past 30 days	76 (82.6)

SD, Standard Deviation; EDS, Everyday Discrimination Scale.

All participants reported experiencing at least one microaggression during their lifetime (Supplementary Table 1). The most frequently endorsed items were “You are treated with less respect than other people are” (96.7%), “You are treated with less courtesy than other people are” (94.6%),” and “People act as if they are better than you are” (94.6%). A total of 82.6% of participants reported experiencing microaggressions in the past 30 days. The most common included: “People act as if they think you are not smart (69.8%),” “People act as if they are better than you are (57.6%),” and “You are treated with less courtesy than other people are (52.2%).” Two items showed sex differences in lifetime endorsement: “People act as if they are afraid of you” was reported by 79.6% of women versus 93.6% of men [χ^2^ (1, *N* = 92) = 3.93, *p* = 0.04], and “You are threatened or harassed” was reported by 79.6% of women versus 55.3% of men [χ^2^ (1, *N* = 92) = 6.04, *p* = 0.01].

Results of the Cochran–Mantel–Haenszel and Logit models predicting past-30-day binge drinking as a function of past-30-day microaggressions, depression, and anxiety are presented in Table 2. Although none of the associations reached statistical significance, we observed a trend indicating that depression may relate to higher odds of binge drinking among participants reporting recent microaggression experiences (common OR = 2.34, 95% CI: 0.92, 5.98; Cohen’s *d* = 0.48). Stratified analyses indicated that this trend was driven by individuals who had experienced microaggressions in the past 30 days. Moderate/severe depression was associated with a trend toward elevated odds of binge drinking in this group (OR = 2.58, 95% CI: 0.94, 7.01, Cohen’s d = 0.52).

**Table 2:** Results of Cochran–Mantel–Haenszel and logit models predicting any past-30-day binge drinking as a function of past-30-day microaggressions, depression, and anxiety.

Predictor	Stratification variable		OR (95% CI) for binge drinking P30D	*p*-value
Depression (Ref: None/Mild)	Past-30-day microaggressions	No	1.25 (0.09, 17.65)	
		Yes	2.58 (0.94, 7.01)	
	*Common*		2.34 (0.92, 5.98)	0.07
Anxiety (Ref: None/Mild)	Past-30-day microaggressions	No	0.55 (0.03, 10.93)	
		Yes	1.80 (0.67, 4.84)	
	*Common*		1.60 (0.63, 4.07)	0.32
Past-30-day microaggressions (Ref: No)	Depression	None/mild	0.89 (0.24, 3.31)	
		Moderate/severe	1.83 (0.15, 22.58)	
	*Common*		1.03 (0.32, 3.30)	0.96
Past-30-day microaggressions (Ref: No)	Anxiety	None/mild	0.93 (0.25, 3.42)	
		Moderate/severe	3.00 (0.17, 52.10)	
	*Common*		1.11 (0.34, 3.64)	0.85
Past-30-day microaggressions (Ref: No)	Sex	Female	0.96 (0.20, 4.52)	
		Male	1.97 (0.38, 10.30)	
	*Common*		1.33 (0.43, 4.08)	0.62

OR, Odds Ratio; CI, Confidence Interval; Ref, Reference. All Breslow-Day tests for homogeneity of the odds ratios >0.05, indicating the existence of common odds ratio.

## Discussion

This preliminary study revealed the pervasiveness and range of microaggressions experienced among Black young adult regular drinkers. Over half reported being treated as if they are not as intelligent or as good as others or being treated with less courtesy than other people in the past 30 days. This exploratory investigation also revealed differences by sex in the prevalence of certain microaggressions, with harassment or threats more common among females and people acting afraid of [the participant] more common among males. Inconsistent with the larger literature (6, 7), we did not find a direct association between microaggressions and binge drinking. However, our exploratory findings suggest a possible pattern in which depression could be linked to binge drinking among Black young adults who recently experienced microaggressions. Although these associations were not statistically significant, the direction and magnitude of effects indicate a potential contextual influence that should be examined in larger, adequately powered studies. That is, microaggressions may play an important contextual role in shaping binge drinking behaviors among Black young adults experiencing depression.

The well-established link between problematic alcohol use and depression (24) is consistent with the self-medication hypothesis that alcohol can be used as a means of coping with negative affect (25). The observed elevated likelihood of binge drinking among participants experiencing depression who also recently experienced microaggressions underscores how the heightened mental distress associated with microaggressions further increases the likelihood of using alcohol to manage negative affect (26). Findings from research demonstrating mediating effects of depression on the association between microaggressions and substance use in Black young adults (27) also suggest that this is a pathway of risk. These findings could inform the development of future responsive ecological momentary interventions for problem drinking among Black young adults. They suggest the importance of including content related to managing negative affect and the development of alternative coping skills, as well as the delivery of just-in-time prompts to engage in these alternative skills after recent experiences of microaggressions to reduce binge drinking instances. This observed intersection between mental health and alcohol use underscores the urgent need for culturally responsive interventions to address these interconnected challenges, particularly in Black young adults.

### Limitations

There are certain limitations of the current study. As a pilot study with a modest sample size, our analyses had limited power to detect small to moderate associations. Non-significant CMH tests should therefore be interpreted as inconclusive rather than as evidence of no association. Additionally, as EDS items were not specific to microaggressions based on race, endorsement may have been higher among participants with other marginalized identities (e.g., transgender, genderqueer). Relatedly, some of the more overt acts of discrimination assessed in the EDS may not be considered microaggressions under every definition. There also may have been some experiences of microaggressions among Black young adults that the 9 EDS items used in this study did not capture. Future research should consider garnering feedback from Black young adults on subtle and nuanced ways in which microaggressions come up in their daily lives, to inform the addition of EMA items which may more comprehensively capture the unique lived experiences and needs of this group. Further, as statistical power would have been insufficient to assess gender differences for certain categories of gender, we used sex as a proxy in stratified analyses. In addition, although social media use is nearly ubiquitous among young adults, the limitation of recruitment to social media users should be considered. Finally, given the pilot nature of this study and our relatively small sample size, we were likely underpowered to detect some key associations (e.g., between microaggressions and alcohol use). Likewise, as an exploratory pilot study designed to lay the groundwork for an EMA study, we did not adjust for multiple testing.

## Conclusion

Our findings support the importance of assessing depression and microaggressions in relation to alcohol use in real time, a focus of the final, EMA phase of this pilot study. The granular, moment-to-moment data provided by EMA will enhance our understanding of the role microaggressions play. These critical insights will inform the design of future mHealth interventions targeting depression and alcohol use among Black young adults engaged in problem drinking, expanding the impact of existing digital interventions by incorporating content and personalized communications, which is often preferred by Black young adults (28).

## Data Availability

The raw data supporting the conclusions of this article will be made available by the authors, without undue reservation.
