# Supplementary material for: Associations between microaggressions, depression, anxiety, and alcohol use among Black young adults: findings from a pilot study
Source: Front Public Health. 2026 Jan 15;13:1694000. doi: 10.3389/fpubh.2025.1694000 (PMC12852343; doi:10.3389/fpubh.2025.1694000)
Supplement: Supplementary file 1 [file Table_1.docx]

| Supplementary Table 1. Proportion of participants endorsing Everyday Discrimination Scale items: lifetime and past 30 days | | | | | | |
| --- | --- | --- | --- | --- | --- | --- |
|  | Lifetime | | | Past 30 days | | |
|  | Full sample | Female | Male | Full sample | Female | Male |
|  | N (%) | | | | | |
| Any discrimination experience | 92 (100.0) | 47 (100.0) | 45 (100.0) | 76 (82.6) | 35 (79.6) | 41 (85.1) |
| *Everyday Discrimination Scale Items* |  |  |  |  |  |  |
| You are treated with less courtesy than other people are | 87 (94.6) | 41 (93.2) | 45 (95.7) | 48 (52.2) | 23 (62.3) | 24 (51.1) |
| You are treated with less respect than other people are | 89 (96.7) | 42 (95.5) | 46 (97.9) | 45 (48.9) | 21 (47.7) | 23 (48.9) |
| You receive poorer service than other people at restaurants or stores | 82 (89.1) | 40 (90.9) | 42 (89.4) | 31 (33.7) | 18 (40.9) | 13 (27.7) |
| People act as if they think you are not smart | 85 (92.4) | 42 (95.5) | 42 (89.4) | 55 (59.8) | 27 (61.4) | 27 (57.5) |
| People act as if they are afraid of you | 80 (87.0) | **35 (79.6)** | **44 (93.6)** | 46 (50.0) | 20 (45.5) | 26 (55.3) |
| People act as if they think you are dishonest | 79 (85.9) | 36 (81.8) | 44 (93.6) | 32 (34.8) | 12 (27.3) | 19 (40.4) |
| People act as if they're better than you are | 87 (94.6) | 42 (95.5) | 26 (55.3) | 53 (57.6) | 28 (63.6) | 24 (51.1) |
| You are threatened or harassed | 62 (67.9) | **35 (79.6)** | **26 (55.3)** | 11 (12.0) | 7 (15.9) | 3 (6.4) |
| You are called names or insulted | 75 (81.5) | 36 (81.8) | 38 (80.9) | 22 (23.9) | 10 (22.7) | 11 (23.4) |
| **Bold** indicates statistically significant at *p<*0.05. |  |  |  |  |  |  |
